# Rates of Variants of Uncertain Significance Among Patients With Breast Cancer Undergoing Genetic Testing: Regional Perspectives

**DOI:** 10.3389/fonc.2022.673094

**Published:** 2022-03-25

**Authors:** Hikmat Abdel-Razeq, Faris Tamimi, Lama Abujamous, Rashid Abdel-Razeq, Mahmoud Abunasser, Sara Edaily, Hazem Abdulelah, Razan Abu Khashabeh, Rayan Bater

**Affiliations:** ^1^ Department of Medicine, King Hussein Cancer Center, Amman, Jordan; ^2^ School of Medicine, University of Jordan, Amman, Jordan; ^3^ Department of Cell Therapy & Applied Genomic, King Hussein Cancer Center, Amman, Jordan; ^4^ Department of Internal Medicine, Istishari Hospital, Amman, Jordan

**Keywords:** breast cancer, genetic testing, BRCA, variants of uncertain significance, VUS

## Abstract

**Purpose:**

Contrary to BRCA pathogenic variants, recommendations for management of variants of uncertain significance (VUS) are not clear and focus more on the patient’s family and personal history of cancer. Local and regional data on VUS are scarce. In this paper, we study patterns and frequency of VUS among breast cancer patients undergoing genetic testing.

**Patients and Methods:**

Patients with breast cancer at high risk for pathogenic variants, as per the National Comprehensive Cancer Network (NCCN) guidelines, were tested at reference laboratories. Related surgical interventions were reviewed.

**Results:**

Among a group of 1,197 patients with breast cancer who underwent genetic testing and counseling, 110 (9.2%) had VUS; most (n = 79, 71.8%) were in *BRCA2*. Median age (range) was 39 (25–66) years with 65 (59.1%) patients who were 40 years or younger at diagnosis. Among 103 patients with non-metastatic disease, 48 (46.6%) had breast-conserving surgery (BCS) while only 5 (4.9%) had bilateral mastectomies; all were due to bilateral disease and not prophylactic. VUS diagnosis was known prior to initial surgery in 34 (33.0%) patients; 11 (32.4%) of them had BCS only. Over the study period, only one VUS variant was upgraded to “likely positive.” The recent introduction of multiple-gene panel testing had resulted in a surge in VUS rate (22.2%) in genes other than *BRCA1* or *BRCA2*, like *PALB2, CHEK2*, and *ATM.*

**Conclusions:**

Rates of VUS are relatively high and increasing, mostly in non-*BRCA1* or *BRCA2*, and this had no impact on the therapeutic or prophylactic surgical decisions. Adherence to guidelines is extremely important to avoid unnecessary procedures.

## Introduction

Breast cancer is the most common cancer among women in Jordan and worldwide (1, 2). Almost 10% of breast cancer cases are hereditary and mostly related to *BRCA1* or *BRCA2* gene mutations (3, 4); both were initially characterized and sequenced more than 25 years ago (5, 6) and since then have become the most thoroughly studied genes in cancer biology (7).

*BRCA1* or *BRCA2* mutations are associated with high penetrance rates; the estimated mean cumulative risks for breast cancer by age 70 years for *BRCA1* and *BRCA2* mutation carriers, as reported by a meta-analysis, were 57% (95% CI, 47% to 66%) and 49% (95% CI, 40% to 57%), respectively (8). Such patients are at a higher risk for ovarian cancer, too (9). Risk-reduction interventions, such as bilateral mastectomies and oophorectomies, are highly recommended for such patients (10).

Genetic test results are usually reported as positive (pathogenic, or likely pathogenic mutation), negative (no detected mutation), or a variant of uncertain significance (VUS). The latter is a DNA alteration in the gene sequence with unknown consequences on the gene function (11).

Contrary to pathogenic mutations, recommendations for the management of VUS are not clear and focus more on clinical factors and personal and family history of cancer—breast and ovarian in particular. Genetic variants that may or may not have clinical consequence can be confusing and anxiety-provoking to patients and physicians alike (12).

The frequency of VUS reports varies worldwide and depends on the ancestry of the population served, testing prevalence, and methodology; rates as high as 21% were reported among the African-American population (13). The increased public awareness, sparked by media reporting of celebrities, resulted in growing interest by the public about the genetic background of cancer in general, and breast cancer in particular (14, 15). Additionally, the recent linkage between triple-negative breast cancer, with or without family history, and *BRCA* mutations along with the recent introduction of novel targeted therapies for such a subgroup of patients have also increased the requests for genetic testing (16–19).

No data on VUS exist on Arab patients in general, and Jordanians in particular. In this study, we search for the frequency of VUS among breast cancer patients and study treatment patterns and risk-reduction interventions related to such findings.

## Methods

In 2016, we started a genetic testing and genetic counseling clinic for patients with breast cancer. Patients were referred to this service if they fit one of the indications recognized by the National Comprehensive Cancer Network (NCCN) guidelines (20). Such indications include patients with early-onset breast cancer (age ≤40 years), aged ≤60 years with triple-negative disease, and those diagnosed at age 50 years or younger with one or more close relatives with pancreatic, prostate (Gleason score ≥7), or breast cancer at any age. Additionally, patients diagnosed at any age with one or more close relatives with breast cancer diagnosed at age 50 years or younger and those diagnosed at any age with two or more additional diagnoses of breast cancer at any age were also tested. Since then, the database contains records on almost 1,200 patients and data on pathogenic variants were reported earlier (21, 22).

We also utilized patients’ medical records to review clinical and pathological features of their breast cancer along with surgical interventions patients had in relation to their breast cancer and VUS identification.

Eligible patients were identified during their routine oncology clinic visits or during the weekly breast multidisciplinary team (MDT) meetings. A detailed three-generation family history was also obtained by a genetic counselor or one of the investigators. Ten milliliters of peripheral blood samples was obtained for DNA extraction. *BRCA1* and *BRCA2* sequencing was performed at one of two reference laboratories: Leeds Cancer Center, Leeds, United Kingdom, and Myriad Genetics Laboratory, Salt Lake City, USA. In November 2019, we expanded our genetic testing to include a panel of 20 genes (*ATM, BARD1, BRCA1, BRCA2, BRIP1, CDH1, CHEK2, EPCAM, MLH1, MSH2, MSH6, NBN, NF1, PALB2, PMS2, PTEN, RAD51C, RAD51D, STK11, TP53*) and samples were tested at Invitae, San Francisco, USA. Consent for genetic testing was obtained following a detailed explanation and interview by a trained genetic counselor. The study was approved by King Hussein Cancer Center’s Institutional Review Board (IRB).

### Statistical Analysis

Descriptive data of clinical and pathological characteristics of patients were collected, tabulated, and described by ranges, medians, and percentages (%). The chi-square test was used to compare the proportion of VUS according to age (≤40 versus >40), triple-negative status, and family history, in which a p-value of ≤ 0.05 was considered significant. Relatives diagnosed with breast cancer and tested after the index case in the family were not enrolled and were excluded from the analysis. The Fisher exact test was used to compare the type of surgical management; modified radical mastectomy (MRM) vs. BCS according to timing of genetic testing; and before surgery or after surgery. Analyses were conducted using Minitab^®^ Statistical Software version 18 (2017), Minitab Inc., State College, USA.

## Results

Between May 2016 and May 2020, a total of 1,197 patients were tested for *BRCA1* and *BRCA2* mutations and were included in our genetic testing database, and all are Jordanians. Patients were referred for testing as per the NCCN guidelines (20). The most common indication for testing were age ≤ 40 years (n = 645, 53.9%) and positive family history of breast, pancreatic, or high-grade prostate cancer (n = 593, 49.5%). Positive/likely positive variants were detected in 143 (12.0%) patients.

Among the whole group, 110 (9.2%) patients had VUS in *BRCA1* or *BRCA2* genes and are the subject of this study. Except for one patient, all were women and the median age (range) was 39 (25–66) years with 65 (59.1%) who were 40 years or younger at diagnosis. At the time of genetic counseling and testing, all patients had current or recent history of breast cancer; details of patients’ characteristics are presented in **Table 1**.

**Table 1:** Patients’ characteristics (n = 110).

Characteristics		Number	(%)
Age at diagnosis (years)	Median	39	
	Range	25–66	
Hormonal status	ER-positive	81	73.6
	PR-positive	85	77.3
HER-2 status	HER2-positive	21	19.1
	HER2-negative	86	78.2
	Unknown	3	2.7
Triple negative		13	11.8
Disease stage	Early stage	103	93.6
	Metastatic	7	6.4

ER, estrogen receptor; PR, progesterone receptor; HER2, human epidermal growth factor receptor 2.

Among the patients with VUS, 13 (11.8%) had triple-negative disease while 12 (10.9%) others had bilateral or two or more unilateral primary breast cancers. Family history of breast, ovarian, or pancreatic cancers, in at least one close relative, was identified in 51 (46.4%) patients. Most VUS (n = 79, 71.8%) were in *BRCA2*, and 18 (16.4%) had two (n = 14) or 3 (n = 4) different variants. Rates of VUS were not different in relation to age, presence of triple-negative disease, family history of breast, ovarian, or prostate cancers, and those with bilateral or more than one unilateral breast cancers (**Table 2** **)**.

**Table 2:** VUS rates in relation to indications for genetic testing (n = 1,197).

Indication for genetic testing		Number of patients	VUS Number (rate)	p-value
Age at diagnosis (years)	≤40	645	65 (10.1%)	0.294
	>40	552	45 (8.2%)	
Triple-negative, age ≤ 60 years	Yes	157	13 (8.3%)	0.699
	No	1040	97 (9.3%)	
Diagnosed at age ≤50 years with ≥1 close relative with breast cancer at any age, ≥1 close relative with pancreatic cancer, or ≥1 close relative with prostate cancer (Gleason score ≥7)	Yes	593	51 (8.6%)	0.524
	No	604	59 (9.8%)	
Diagnosed at any age with ≥1 close relative with breast cancer diagnosed at age 50 years or younger	Yes	339	29 (8.6%)	0.662
	No	858	81 (9.4%)	
Diagnosed at any age with two or more additional diagnosis of breast cancer at any age (synchronously or asynchronously)	Yes	151	12 (8.0%)	0.604
	No	1046	98 (9.4%)	

**Figure 1:**
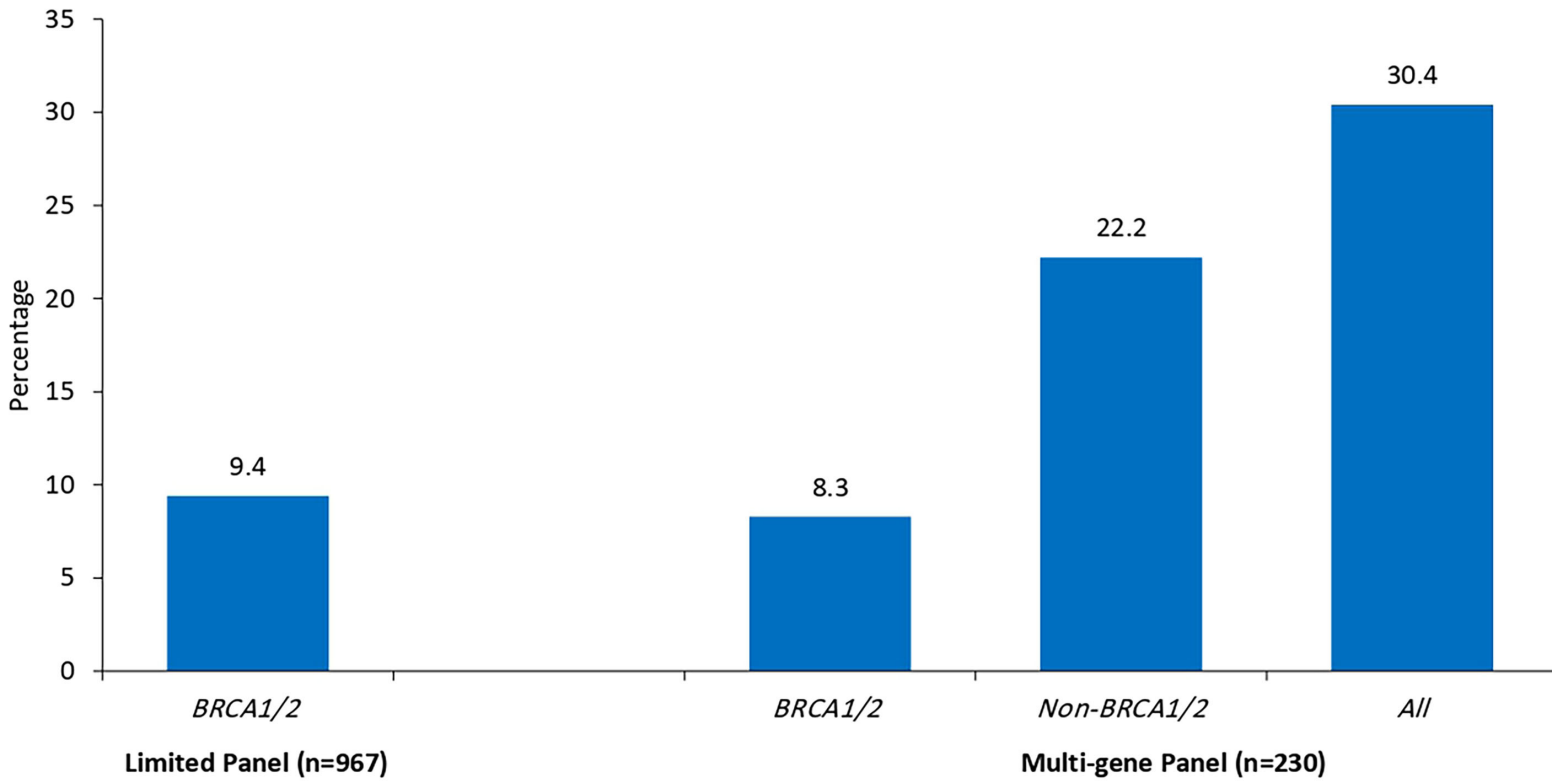
Rates of Variants of Uncertain Significance (VUS) by testing panel (n = 1197).

Since we started genetic testing 4 years ago, we have not witnessed significant changes in VUS rates. The classification of variants was upgraded from VUS to likely positive in only one patient (*BRCA1* exon 5–8 duplications). The recent introduction of multiple-gene panel testing resulted in a surge of VUS rates in genes other than *BRCA1* or *BRCA2*. Among the whole group, a total of 230 patients were tested using the recently introduced multigene panels, and positive/likely positive variants were detected in 27 (11.7%) patients; 14 (51.9%) were in genes other than *BRCA1* and *BRCA2*. Additionally, VUS variants in *BRCA1* or *BRCA2* genes were detected in 19 (8.3%) patients while 51 (22.2%) others had VUS in many genes other than *BRCA1* or *BRCA2*; 19 (37.3%) of them had 2 (n = 16) or 3 (n = 3) various VUS. The most common mutations encountered were in *PALB2, CHEK2, ATM, BARD1, NBN*, and *NF1*. **Figure 1** details VUS rates using the limited two-gene panel (*BRCA1* and *BRCA2*) versus a multigene (20-gene) panel. Variant types and classification encountered are detailed in supplementary tables (**Table 3** for *BRCA1/2* variants and **Table 4** for non-*BRCA1/2* variants).

**Table 3:** Variants of Uncertain Significance (VUS) Mutations in *BRCA1* and *BRCA2*.

Gene	Nucleotide change	Amino acid change	Variant type	Frequency
BRCA2	c.9875C>T	p.Pro3292Leu	missense	4
BRCA2	c. 9613_9614delinsCT	p.Ala3205Leu	missense	4
BRCA2	c.62A>G	p.Lys21Arg	missense	2
BRCA2	c.502C>G	p.Pro168Ala	missense	2
BRCA2	c.4621A>C	p.Lys1541Gln	missense	2
BRCA1	c.994C>T	p.Arg332Trp	missense	2
BRCA2	c.4252A>G	p.Ile1418Val	missense	3
BRCA2	c.865A>G	p.Asn289Asp	missense	2
BRCA2	c.5126A>C	p.Asp1709Ala	missense	2
BRCA1	c.5333-6T>C	NA	Intron Variant	2
BRCA2	c.9586A>G	p.Lys3196Glu	missense	2
BRCA1	c.851A>G	p.Gln284Arg	missense	2
BRCA2	c.8632G>C	p.Glu2878Gln	missense	3
BRCA2	c.8332-3C>G	NA	Intron Variant	2
BRCA2	c.7534C>T	p.Leu2512Phe	missense	4
BRCA2	c.6521T>C	p.Val2174Ala	missense	2
BRCA2	c.8774A>G	p.Gln2925Arg	missense	2
BRCA1	c.2604A>G	p.Ser868=	synonymous	1
BRCA2	c.5976A>G	p.Ser1992=	synonymous	1
BRCA1	c.693G>A	p.Thr231=	synonymous	1
BRCA2	c.122C>T	p.Pro41Leu	missense	3
BRCA2	c.10202C>T	p.Thr3401Met	missense	1
BRCA2	c.7806-6G>T	NA	Intron Variant	1
BRCA2	c.1915T>C	p.=	synonymous	1
BRCA1	c.3367G>T	p.Asp1123Tyr	missense	1
BRCA1	c.4028A>T	p.Asp1343Val	missense	1
BRCA1	c.5555C>T	p.Thr1852Ile	missense	1
BRCA1	c.2123 C>A	p.Ser708Tyr	missense	1
BRCA1	c.5504G>A	p.Arg1835Gln	missense	1
BRCA2	c.2396A>G	p.Lys799Arg	missense	1
BRCA2	c.2366 A>G	p.Leu2366Val	missense	1
BRCA2	c.4943C>T	p.Ala1648Val	missense	1
BRCA1	c.4185+10G>A	NA	Intron Variant	1
BRCA2	c.7462A>G	p.Arg2488Gly	missense	1
BRCA2	c.8755-19 A>G	NA	Intron Variant	2
BRCA2	c.3676A>C	p.Lys1226Gln	missense	2
BRCA2	c.10078A>G	p.Lys3360Glu	missense	1
BRCA1	c.4412G>A	p.Gly1471Asp	missense	1
BRCA2	c.5897A>G	p.His1966Arg	missense	2
BRCA1	c.4045A>C	p.Thr1349Pro	missense	1
BRCA2	c.2332G>C	p.Val778Ile	missense	1
BRCA2	c.277T>G	p.Ser93Ala	missense	1
BRCA2	c.5590G>A	p.Asp1864Asn	missense	1
BRCA1	c.5074+6 C>G	NA	Intron Variant	1
BRCA1	c.478G>A	p.Gly160Arg	missense	1
BRCA2	c.1769T>G	p.Phe590Cys	missense	1
BRCA2	c.1793C>T	p.Thr598Ile	missense	1
BRCA2	c.9839C>A	p.Pro3280His	missense	1
BRCA2	c.280 C>T	p.Pro94Ser	missense	1
BRCA2	c.9754_9765del	p.Ser3252_Gly3255del	deletion	1
BRCA2	c.800G>A	p.Gly267Glu	missense	1
BRCA1	c.3149G>C	p.Gly2748Asp	missense	1
BRCA1	c.539T>C	p.Ile180Thr	missense	1
BRCA1	c.3587C >T	p.Thr1196Ile	missense	2
BRCA2	c.4954G>A	p.Ala1652Pro	missense	1
BRCA1	c.3642G>T	p.Glu1214Asp	missense	1
BRCA2	c.7301A>C	p.Lys2434Thr	missense	1
BRCA1	exon 5-8 duplication	NA	duplication	1
BRCA2	c.1550A>G	p.Asn517Ser	missense	1
BRCA2	c.9502-12T>G	NA	Intron Variant	1
BRCA2	c.6986 C>T	p.Pro2329Leu	missense	1
BRCA2	c.752C>G	p.Thr251Arg	missense	1
BRCA1	c.3526 G>A	p.Val1176Ile	missense	1
BRCA1	c.1662G>C	p.Glu554Asp	missense	1
BRCA2	c.2072 C>T	p.Ala691Val	missense	1
BRCA2	c.9378G>C	p.Gln3126=	synonymous	1
BRCA1	c.509G>A	p.Arg170Gln	missense	1
BRCA2	c.9781G>A	p.Asp3261Asn	missense	1
BRCA2	c.1263 A>G	p.=	synonymous	1
BRCA1	c.1446_1448del	p.Ile483del	missense	1
BRCA2	c.4594G>T	p.Val1532Phe	missense	1
BRCA2	c.9418G>A	p.Ala3140Thr	missense	1
BRCA2	c.2779A>G	p.Met927Val	missense	1
BRCA1	c.4357+5G>A	NA	Intron Variant	1
BRCA2	c.7992T>G	p.Ile2664Met	missense	1
BRCA2	c.644_646del	p.Glu215del	deletion	1
BRCA1	Exons 1-2 Duplication	NA	duplication	1
BRCA2	c.6945 A>G	p.Ile2315Met	missense	1
BRCA2	c.340C>T	p.His114Tyr	missense	1
BRCA2	c.8382C>G	p.Phe2794Leu	missense	1
BRCA2	c.1691C>G	p.Pro564Leu	missense	1
BRCA1	c.1333G>C	p.Glu445Gln	missense	1
BRCA2	c.6916G>C	p.Ala2306Pro	missense	1
BRCA1	c.4434G>C	p.Glu1478Asp	missense	1
BRCA2	c.6805G>A	p.Gly2748Asp	missense	1

Many patients had more than one variant, so total number of variants is more than the total number of patients.

p.= means the entire protein coding region was analyzed and no variant was found that changes (or is predicted to change) the protein sequence.

**Table 4:** Variants of Uncertain Significance (VUS) Mutations in Non-*BRCA1* or *BRCA2*.

Gene	Nucleotide change	Amino acid change	Variant type	Frequency
ATM	c.5892 G>C	p.Lys1964Asn	missense	1
ATM	c.37C>T	p.Arg13Cys	missense	1
ATM	c.8712G>C	p.Glu2904Asp	missense	1
ATM	c.4421 A>G	p.His1474Arg	missense	1
ATM	c.6733G>C	p.Glu2245Gln	missense	1
ATM	c.6975G>A	p.Ala2325=	synonymous	1
ATM	c.133C>T	p.Arg45Trp	missense	2
BARD1	c.59C>T	p.Pro20Leu	missense	1
BARD1	c.1108C>T	p.Arg370Cys	missense	1
BARD1	c.195A<G	p.=	synonymous	1
BARD1	c.1148T>A	p.Met383Lys	missense	1
BARD1	c.247T>C	p.Cys83Arg	missense	1
BARD1	c.1267A>G	p.Lys423Glu	missense	1
BARD1	c.1793C>T	p.Thr598Ile	missense	2
BRIP1	c.3178G>A	p.Val1060Ile	missense	1
BRIP1	c.1846A>G	p.Thr616Ala	missense	1
BRIP1	c.1198G>T	p.Asp400Tyr	missense	3
CDH1	c.2369C>T	p.Thr790Ile	missense	1
CDH1	c.1914G>C	p.Trp638Cys	missense	1
CHEK2	c.1556G>T	p.Arg519Leu	missense	1
CHEK2	c.246_260del	p.77_81DQEPE	microsatellite	1
CHEK2	c.886G>T	p.Asp296Tyr	missense	1
CHEK2	c.1336A>G	p.Asn446Asp	missense	1
CHEK2	c.544C>A	p.Pro182Thr	missense	1
CHEK2	c.1216 C>T	p.Arg406Cys	missense	1
CHEK2	c.1570G>A	p.Glu524Lys	missense	1
CHEK2	c.953G>A	p.Arg318His	missense	1
CHEK2	c.592+3A>T	NA	Intron Variant	5
MLH1	c.650G>A	p.Arg217His	missense	1
MLH1	c.919G>A	p.Val307Met	missense	1
MSH2	c.2141C>T	p.Ala714Val	missense	1
MSH2	c.2T>C	p.Met1?	in-frame shift	1
MSH6	c.3029C>G	p.Thr1010Ser	missense	1
MSH6	c.1649C>G	p.Ser550Cys	missense	1
MSH6	c.1774G>A	p.Val592Ile	missense	1
MSH6	c.3104G>A	p.Arg1035Gln	missense	2
NBN	c.602A>G	p.Asp201Gly	missense	1
NBN	c.703-3T	NA	Intron Variant	1
NBN	c.353_355del	p.Ser118del	Deletion	1
NBN	c.1369A>G	p.Asn457Asp	missense	1
NF1	c.1438A>G	p.Lys480Glu	missense	1
NF1	c.1662G>T	p.Gln554His	missense	1
NF1	c.5788C>G	p.Pro1930Ala	missense	1
NF1	c.8431 C>T	p.Arg2811Cys	missense	1
PALB2	c.3296C>G	p.Thr1099Arg	missense	1
PALB2	c.995 T>C	p.Leu332His	missense	1
PALB2	c.3320 T>C	p.Leu1107Pro	missense	1
PALB2	c.1497 G>C	p.Leu499=	synonymous	1
PALB2	c.1748T>G	p.Leu583Trp	missense	1
PALB2	c.1123C>A	p.Leu375Ile	missense	1
PMS2	c.655G>A	p.Gly219Arg	missense	1
PMS2	c.2335G>A	p.Gly779Arg	missense	1
PMS2	c.113C>T	p.Ala38Val	missense	1
PMS2	c.2068A>C	p.Lys690Gln	missense	2
PMS2	c.2012C>T	p.Thr671Met	missense	1
RAD51C	c.431T<C	p.Ile144Thr	missense	2

Many patients had more than one variant, so total number of variants is more than the total number of patients.

p.= means the entire protein coding region was analyzed and no variant was found that changes (or is predicted to change) the protein sequence.

**Figure 2:**
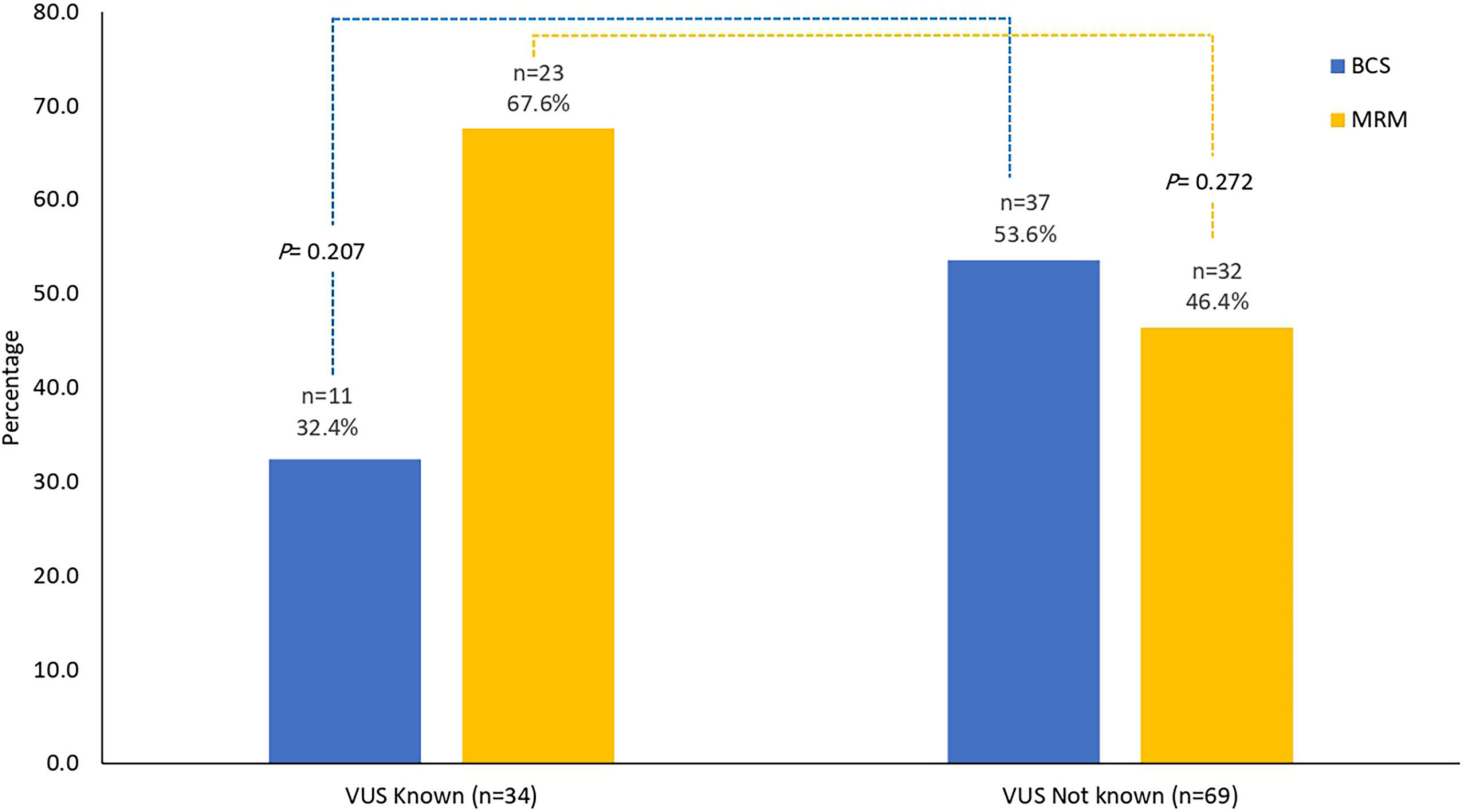
Surgical interventions. Surgical interventions among patients with results of VUS known prior to surgery (n = 34) and those without (n = 69). No difference in the rates of MRM (*p* = 0.272) or BCS (*p* = 0.207). MRM, modified radical mastectomy; BCS, breast-conserving surgery; VUS, variants of uncertain significance.

Among 103 patients with non-metastatic disease, only 5 (4.9%) had bilateral mastectomies; all were due to bilateral disease and not prophylactic. Breast-conserving surgery (BCS) was performed on 48 (46.6%). Genetic testing results (VUS) were known prior to initial surgery in 34 (33.0%) patients; 11 (32.4%) of them had BCS. Rates of MRM and BCS were not different among patients with known VUS prior to the surgery and those without (**Figure 2**) . Only 1 patient of the 103 patients with non-metastatic disease underwent prophylactic oophorectomy.

## Discussion

To our knowledge, this is the first study in Jordan and the region addressing the pattern and frequency of VUS among “Arab” patients who underwent genetic testing following a diagnosis of breast cancer. Our rate of VUS is relatively high, with no indication of getting lower with time. Obviously, the rate of VUS depends on the ancestry of patients as well as the frequency and methodology of testing. Our population is obviously very different from other widely studied ethnic groups, where the rates of VUS are getting significantly lower. In a study conducted by Myriad Genetic Laboratories, Salt Lake City, based on over 20 years of experience and over a million samples tested, the VUS identification rate in both *BRCA1* and *BRCA2* had significantly decreased over the years to rates around 2.0% (23). However, such rates continued to be significantly higher in certain ethnic groups of African, Asian, or Middle Eastern descent (23).

The wider adoption and availability of genetic testing technology, including multiple-gene panels and whole-genome sequencing and at more affordable cost, are expected to keep the rates of VUS even higher especially in nations with diverse ethnicity (24). Such high rates of VUS will likely put lots of pressure on both patients and physicians on risk perception and medical management. This highlights the need to improve our counseling tools and communication skills to better explain these results as misinterpretation of such results can lead to misinformation given to patients, inappropriate medical decisions, and psychological distress for patients and their families (25, 26). Adherence to guidelines is extremely important to avoid unnecessary procedures.

Current international guidelines discourage using VUS results to make major medical decisions like risk-reducing mastectomy or oophorectomy. Instead, more emphasis was put on using patients’ personal history of cancer and close family members to rationalize risk-reducing surgeries (27). Such directions were clearly demonstrated in our patients addressed in this study; none of our patients had risk-reducing surgeries based on VUS findings. Fears of mismanaging such patients are always there as the level of experience of healthcare providers varies significantly. In one study, a survey was sent to 800 United Kingdom (UK) breast cancer specialists to collect data on VUS general knowledge; results interpretation and communication were based on two hypothetical genetics reports. Although 95% of the specialists referred patients for BRCA genetic testing, 71% were not sure about the clinical implications of the test reports presented and 12% never received any genetics training. Better communication of VUS results was reported by specialists when patients under discussion had a positive family history and management guidance was included in the genetic test report. More than a third (39%) did not know how to communicate results to patients with no family history and in cases where reports lacked management guidance (28).

Tools to help physicians better manage these patients exist but are underutilized. Collaborative efforts among commercial labs and academic institutions to study and reclassify VUS from multigene testing resulted in the establishment of an online registry called “Prospective Registry of Multiplex Testing (PROMPT)”; patients, physicians, and institutions can register and follow (29). A similar online regional registry should help our patients, genetic counselors, and physicians better deal with this problem.

## Conclusions

Despite significant decline in VUS rates reported in Western societies, our rate continues to be relatively high and increasing. Our knowledge of VUS had not significantly impacted therapeutic or prophylactic surgical decisions. Such decisions were dictated by patients’ personal and family history. Adherence to guidelines is extremely important to avoid unnecessary procedures.

## Data Availability Statement

The raw data supporting the conclusions of this article will be made available by the authors, without undue reservation.

## Ethics Statement

The studies involving human participants were reviewed and approved by the King Hussein Cancer Center Institutional Review Board (IRB). Written informed consent for participation was not required for this study in accordance with the national legislation and the institutional requirements.

## Author Contributions

Conception and design: HA-R. Provision of study materials or patients: LA, FT, RA, and MA. Collection and assembly of data: LA, FT, RA, HA, SE, and MA. Data analysis and interpretation: HA-R, RB, SE, HA, and RA-R. Manuscript writing: HA-R and RB. Final approval of manuscript: all authors. Accountable for all aspects of the work: all authors. All authors contributed to the article and approved the submitted version.

## Conflict of Interest

The authors declare that the research was conducted in the absence of any commercial or financial relationships that could be construed as a potential conflict of interest.

## Publisher’s Note

All claims expressed in this article are solely those of the authors and do not necessarily represent those of their affiliated organizations, or those of the publisher, the editors and the reviewers. Any product that may be evaluated in this article, or claim that may be made by its manufacturer, is not guaranteed or endorsed by the publisher.
